# Supplementary material for: Soluble St2 Induces Cardiac Fibroblast Activation and Collagen Synthesis via Neuropilin-1
Source: Cells. 2020 Jul 10;9(7):1667. doi: 10.3390/cells9071667 (PMC7408622; doi:10.3390/cells9071667)
Supplement: Supplementary file 1 [file cells-09-01667-s001.pdf]

**Table S1: List of primers used for real-time PCR assays in the manuscript**

| Gene | Primer | Sequence (5´to 3´) |
|---|---|---|
| **NRP-1** | Forward | CTCCTGTTGTGTCTTCAGG |
| | Reverse | CCCGATGAGGATCGGATTC |
| **sST2** | Forward | CGTTACCTTCCTGTGCCATT |
| | Reverse | CTCCATTTGCCAATCATGTG |
| **ST2L** | Forward | AGTTGTGCATTTACGGGAGAG |
| | Reverse | GGATACTGCTTTCCACCACAG |
| **IL-33** | Forward | TGGCCTCACCATAAGAAAGG |
| | Reverse | GCAAACGCTTGGATACTGC |
| **HPRT** | Forward | TTGCTTTCCTTGGTCAGGCA |
| | Reverse | ATCCAACACTTCGTGGGGTC |
| **β-actin** | Forward | GCCGCCAGCTCACCAT |
| | Reverse | TCGATGGGGTACTTCAGGGT |
| **GADPH** | Forward | ACCAGCCCCAGCAAGAGCACAAG |
| | Reverse | TTCAAGGGGTCTACATGGCAACTG |

**Table S2. Proteome alterations induced by sST-2 in human cardiac fibroblasts**

| Protein name | Uniprot code | Gene name | Fold-change ST2 /Control |
|---|---|---|---|
| **Down-regulated proteins in ST-2-treated cells** | | | |
| Histone H1.5 | P16401 | HIST1H1B | 0.56029477 |
| Mitofusin-1 | Q8IWA4 | MFN1 | 0.6570627 |
| Actinfilament-associatedprotein 1 | Q8N556 | AFAP1 | 0.66072435 |
| Charged multivesicular body protein 4a | E9PSI1 | CHMP4A | 0.69763398 |
| SH3 domain-containing kinase-binding protein 1 | Q5JPT2 | SH3KBP1 | 0.70692753 |
| Small nuclear ribonucleoprotein E | P62304 | SNRPE | 0.71390947 |
| Na(+)/H(+) exchange regulatory cofactor NHE-RF2 | Q15599-2 | SLC9A3R2 | 0.73573919 |
| Tumor susceptibility gene 101 protein | F5H442 | TSG101 | 0.75621962 |
| Prefoldinsubunit 2 | Q9UHV9 | PFDN2 | 0.75739442 |
| Synaptobrevinhomolog YKT6 | O15498 | YKT6 | 0.76696515 |
| **Up-regulated proteins in ST-2-treated cells** | | | |
| Nectin-2 | Q92692-2 | PVRL2 | 1.31688694 |
| Eukaryotic translation initiation factor 5B | A0A087WUT6 | EIF5B | 1.32187402 |

| | | | |
|---|---|---|---|
| Neuropilin-1 | E9PEP6 | NRP1 | 1.33730071 |
| E3 SUMO-protein ligase RanBP2 | P49792 | RANBP2 | 1.46901186 |
| ADP-ribosylation factor 5 | P84085 | ARF5 | 1.57810952 |
| Rab-likeprotein 6 | Q3YEC7 | RABL6 | 1.65312128 |
| Interleukin-1 receptor-like 1 | E9PC41 | IL1RL1 | 2.16884842 |
| Ig gamma-1 chain C region | A0A087X1C7 | IGHG1 | 2.28690335 |

## FIGURE LEGENDS

**Supplemental Figure 1.** Representative western blot out of three. Stain free with 9 controls and 8 ST-2 conditions corresponding to 8 datapoints per condition graphed in the manuscript. Complete blots for α-SMA and Collagen 3 from main figure 1A, D.

**Supplemental Figure 2.** Representative western blot out of three. Stain free with 8 controls and 7 ST-2 conditions corresponding to 7 datapoints per condition graphed in the manuscript. Complete blots for Vimentin and CTGF from main figure 1A, F.

**Supplemental Figure 3.** Representative western blot out of two. Stain free with 5 controls and 5 ST-2 conditions corresponding to 5 datapoints per condition graphed in the manuscript. Complete blot for NRP-1 from main figure 2A.

**Supplemental Figure 4.** Representative western blot out of five. Stain free with 4 controls, 4 ST-2 and 4 ST-2+BAY conditions corresponding to 4 datapoints per condition graphed in the manuscript. Complete blots for α-SMA, Vimentin and CTGF from main figure 3B, D.

**Supplemental Figure 5.** Representative western blot out of three. Stain free with 5 controls, 4 ST-2 and 4 ST-2+KO NRP-1 conditions corresponding to 4 datapoints per condition graphed in the manuscript. Complete blots for α-SMA, Vimentin and CTGF from main figure 3H, K.

**Supplemental Figure 6.** Representative western blot out of five. Stain free with 6 controls, 2 NRP-1 $10^{-10}$M, 2 NRP-1 $10^{-9}$M and 2 NRP-1 $10^{-8}$M conditions

corresponding to 2 datapoints per condition graphed in the manuscript. Complete blots for α-SMA and Vimentin from main figure 4A.

**Supplemental Figure 7.** Representative western blot out of two. Stain free with 3 controls, 4 NRP-1 $10^{-10}$M, 4 NRP-1 $10^{-9}$M and 4 NRP-1 $10^{-8}$M conditions corresponding to 4 datapoints per condition graphed in the manuscript. Complete blot for CTGF from main figure 4E.

**Supplemental Figure 8.** Representative western blots out of five from each type. Stain free with 2 controls, 2 conditions of $10^{-8}$M NRP-1 for 5 minutes, 2 conditions of $10^{-8}$M NRP-1 for 10 minutes 2 conditions of $10^{-8}$M NRP-1 for 15 minutes, 2 conditions of $10^{-8}$M NRP-1 for 30 minutes and 2 conditions of $10^{-8}$M NRP-1 for 60 minutes. These blots shown correspond to 2 datapoints per condition graphed in the manuscript. Complete blots for p-NFκB, NFκB, p-p42/44 and p42/44 from main figure 5A, B.

**Supplemental Figure 9.** Representative western blots out of five from each type. Stain free with 2 controls, 2 conditions of $10^{-8}$M NRP-1 for 5 minutes, 2 conditions of $10^{-8}$M NRP-1 for 10 minutes 2 conditions of $10^{-8}$M NRP-1 for 15 minutes, 2 conditions of $10^{-8}$M NRP-1 for 30 minutes and 2 conditions of $10^{-8}$M NRP-1 for 60 minutes. Complete blots for IRAK1/4, p-p38 and p38 from main figure 5C, D.

**Supplemental Figure 10.** Representative western blot out of two. Stain free with 5 controls, 5 NRP-1 and 5 NRP-1+BAY conditions, corresponding to 5 datapoints per condition graphed in the manuscript. Complete blot for α-SMA and Vimentin from main figure 5E.

**Supplemental Figure 11.** Representative western blot out of two. Stain free with 7 controls and 6 PO rats conditions corresponding to 6 datapoints per condition graphed in the manuscript. Complete blots for ST-2, IL-33, MyD88, TRAF6 and AP-1 from main figure 6A, F, G.

**Supplemental Figure 12.** Perivascular myocardial fibrosis in control and PO rats. Immunostaining for α-SMA, vimentin and fibronectin in control and PO rats

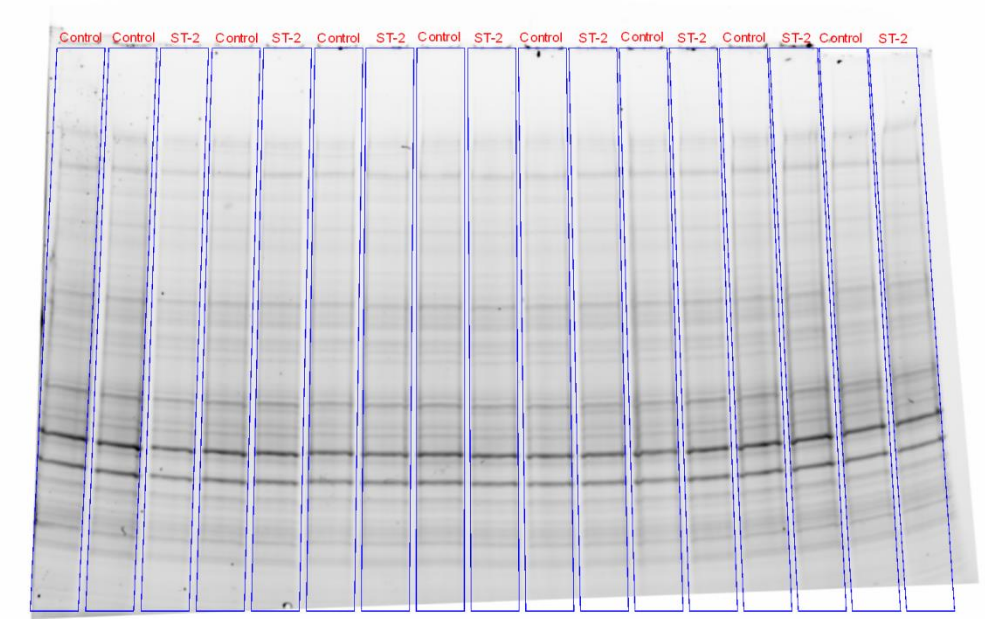


**Figure 1A** - α-SMA – 42 kDa

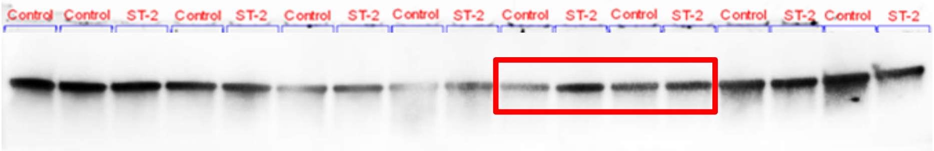


**Figure 1D** - Col3 – 140 kDa

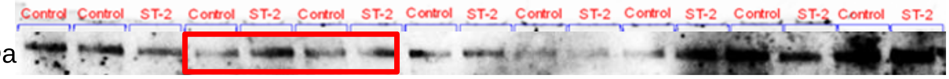


**Supplemental Figure 1**

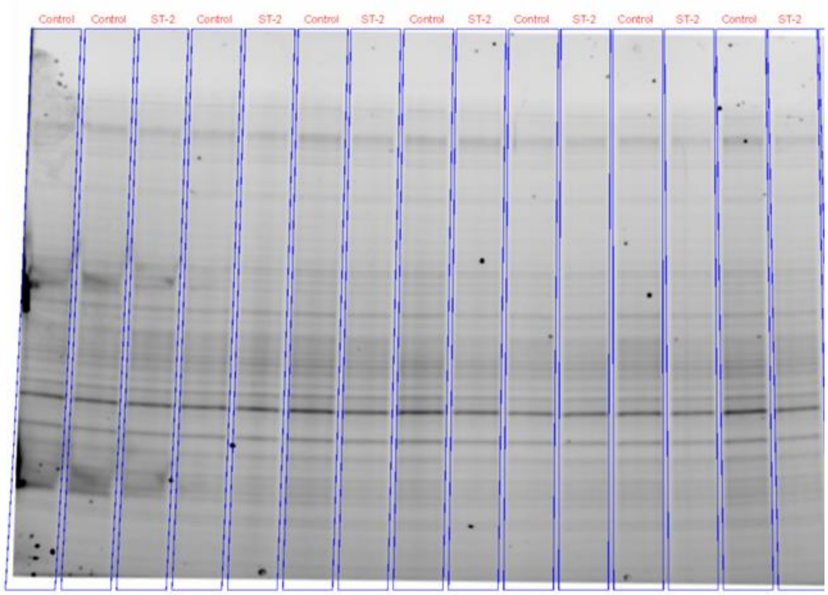


Figure 1A - Vimentin – 54 kDa

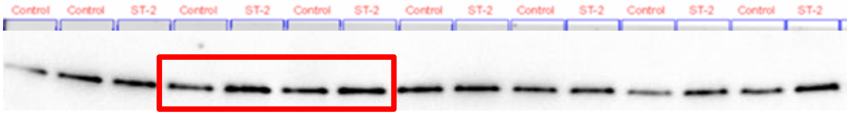


Figure 1F - CTGF– 38kDa

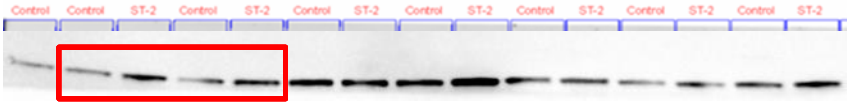


**Supplemental Figure 2**

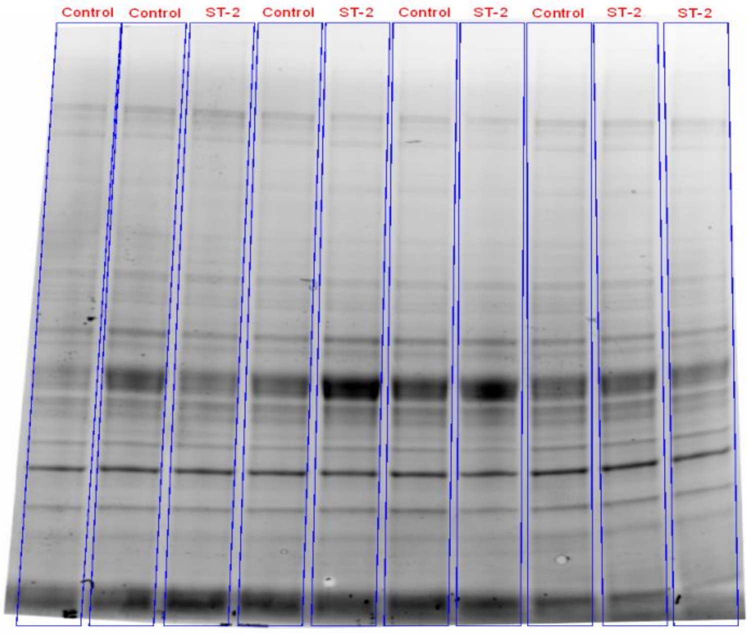


**Figure 2A** - NRP-1 - 103 kDa

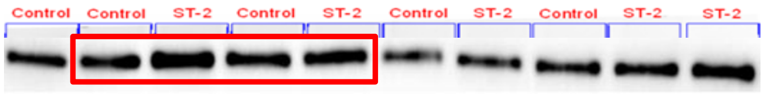


**Supplemental Figure 3**

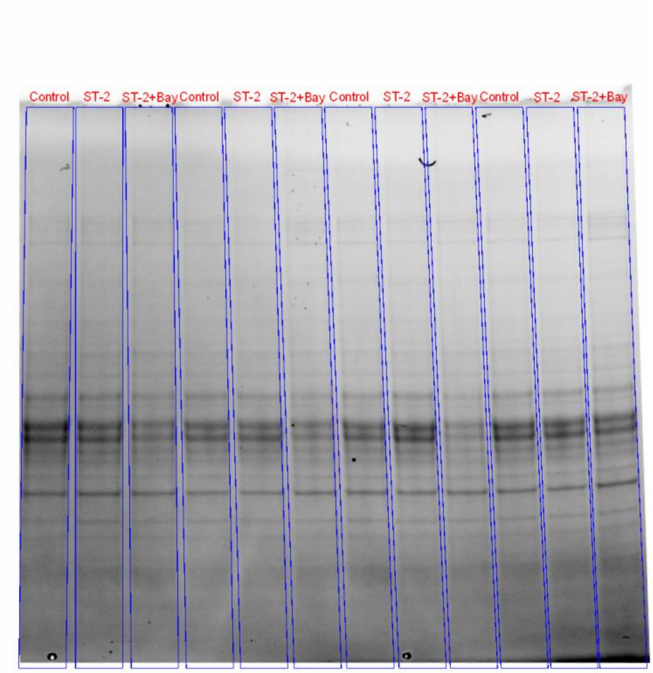


**Figure 3B** – α-SMA – 42 kDa

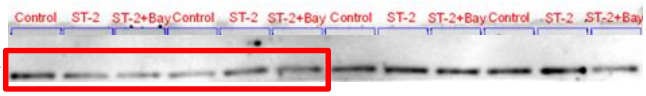


**Figure 3B** - Vimentin – 54 kDa

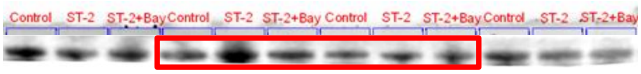


**Figure 3D** - CTGF– 38kDa

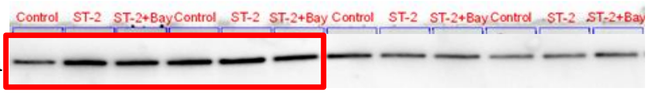


**Supplemental Figure 4**

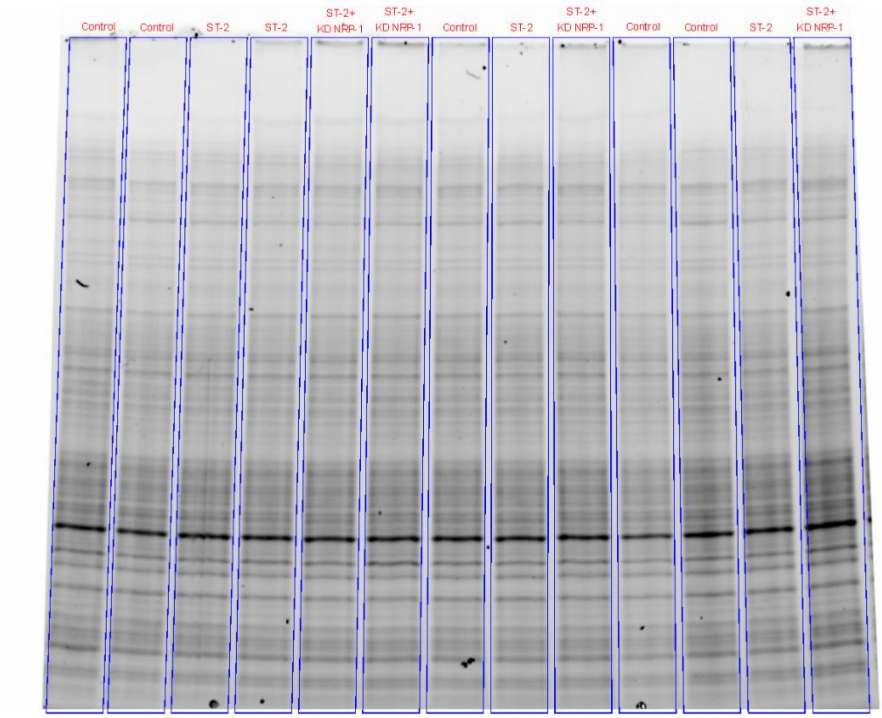


**Figure 3H** - α-SMA – 42 kDa

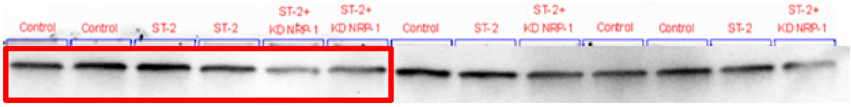


**Figure 3H** - Vimentin – 54 kDa

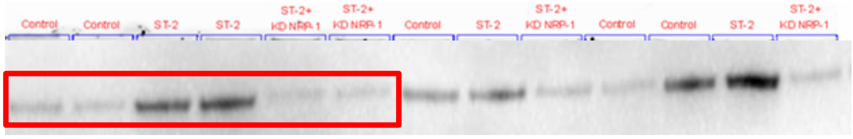


**Figure 3K** - CTGF– 38kDa

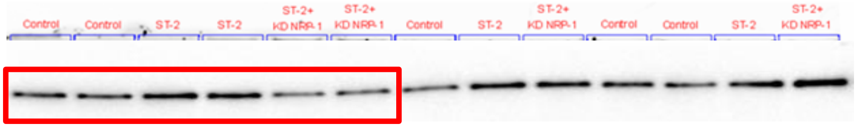


**Supplemental Figure 5**

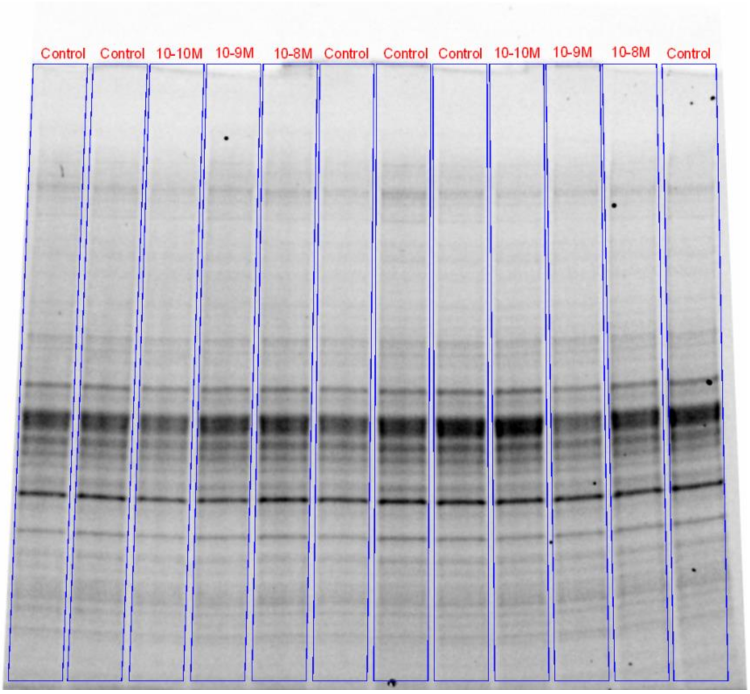


**Figure 4A** - $\alpha$-SMA – 42 kDa

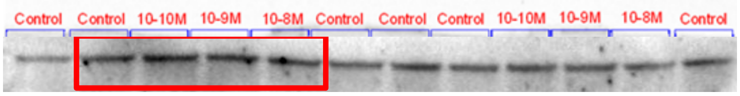


**Figure 4A** - Vimentin – 54 kDa

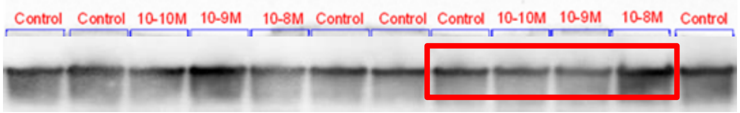


**Supplemental Figure 6**

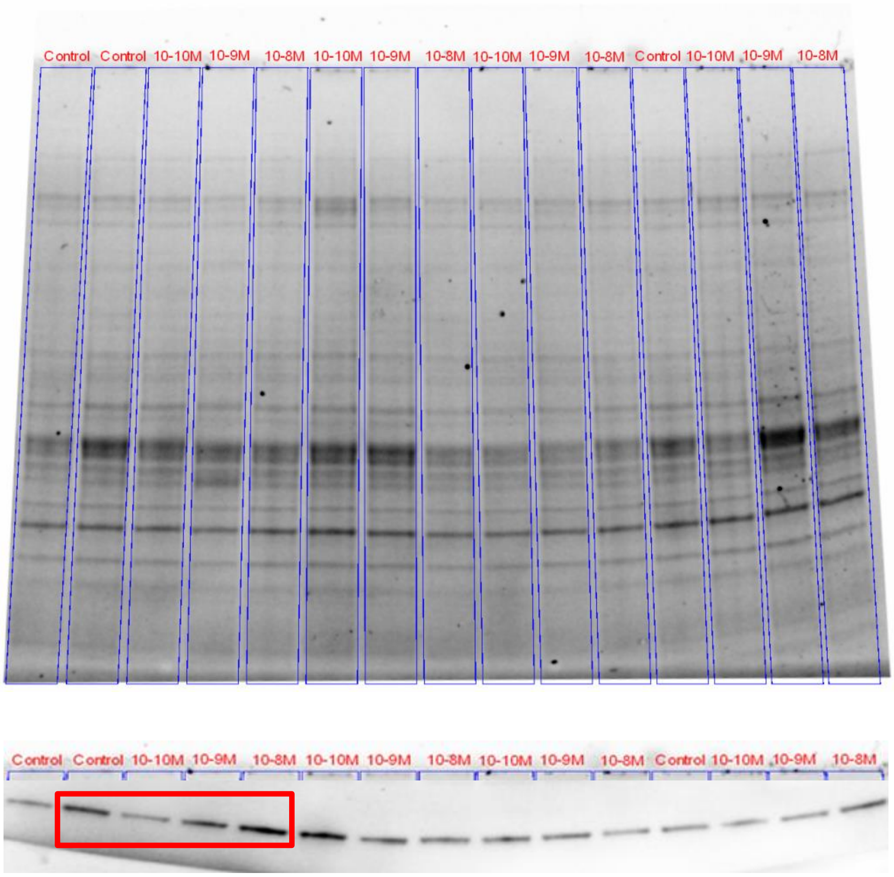


**Figure 4E** - CTGF– 38kDa

**Supplemental Figure 7**

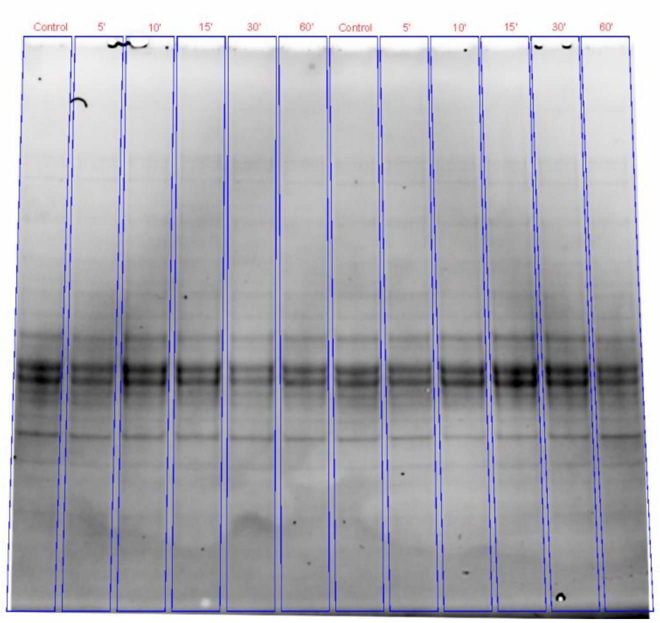


**Figure 5A** - p-NFκB – 65 kDa

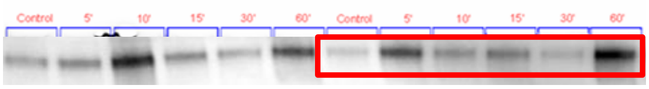


**Figure 5A** - NFκB – 65 kDa

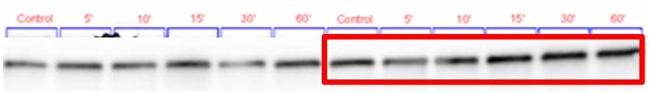


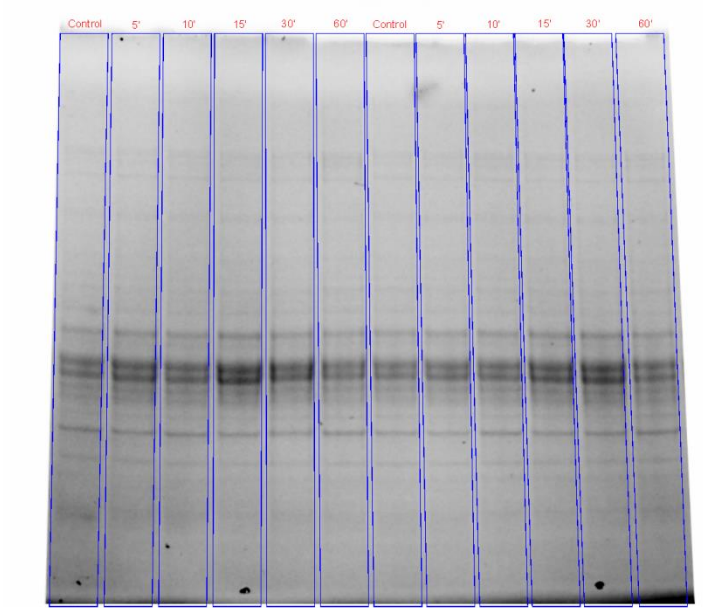


**Figure 5B** - p-p42/44 – 42-44 kDa

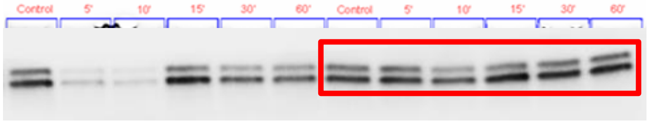


**Figure 5B** - p42/44 – 42-44 kDa

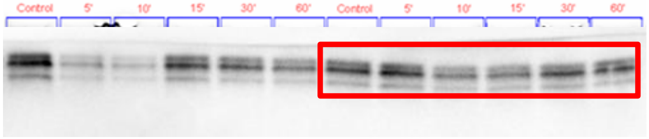


**Supplemental Figure 8**

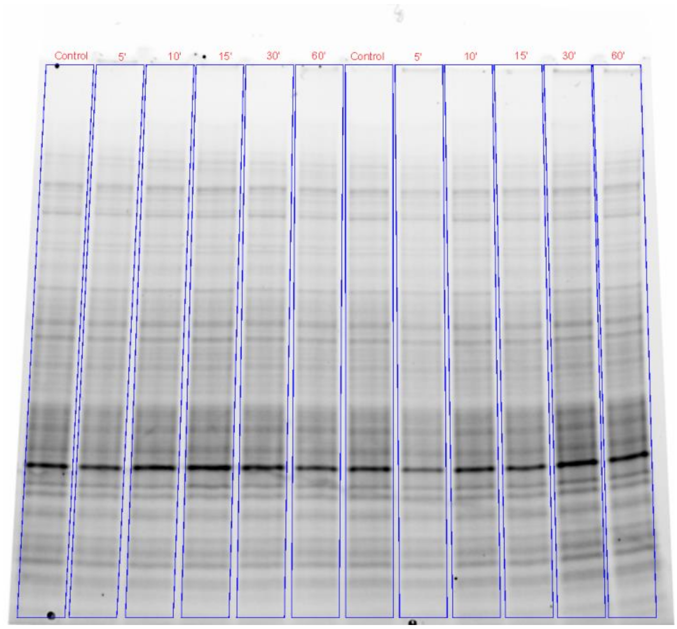


**Figure 5C** - IRAK1/4 – 80 kDa

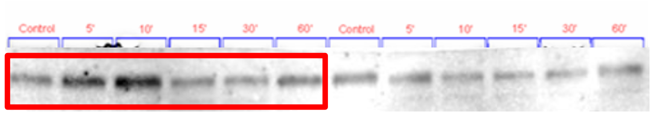


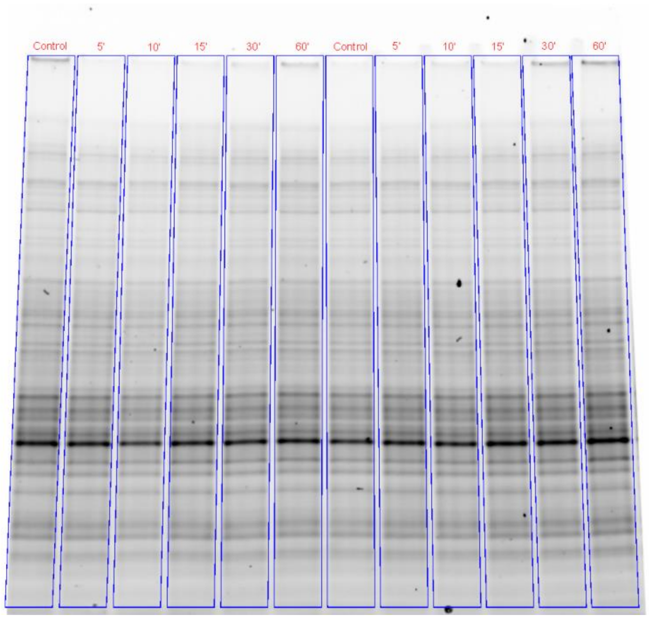


**Figure 5D** - p-p38 – 43 kDa

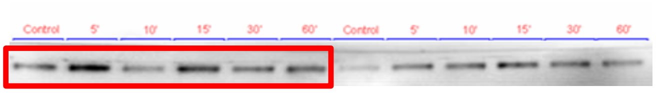


**Figure 5D** - p38 – 43 kDa

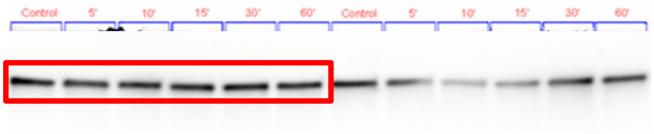


**Supplemental Figure 9**

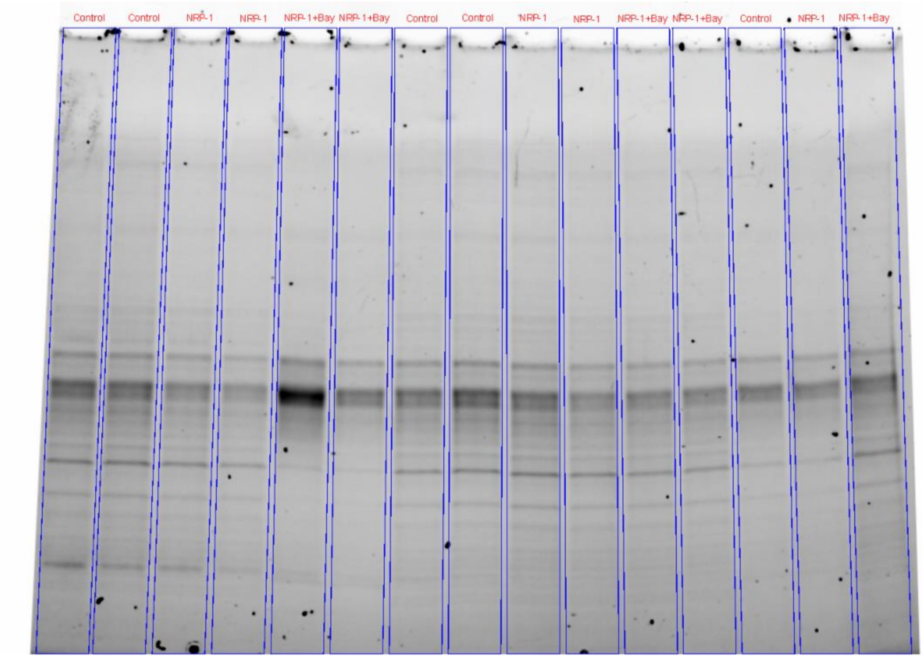


**Figure 5E** - α-SMA – 42 kDa

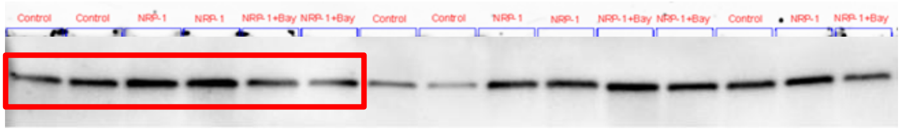


**Figure 5E** - Vimentin – 54 kDa

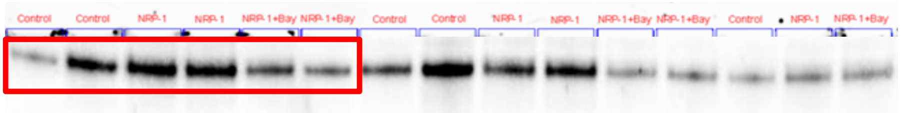


**Supplemental Figure 10**

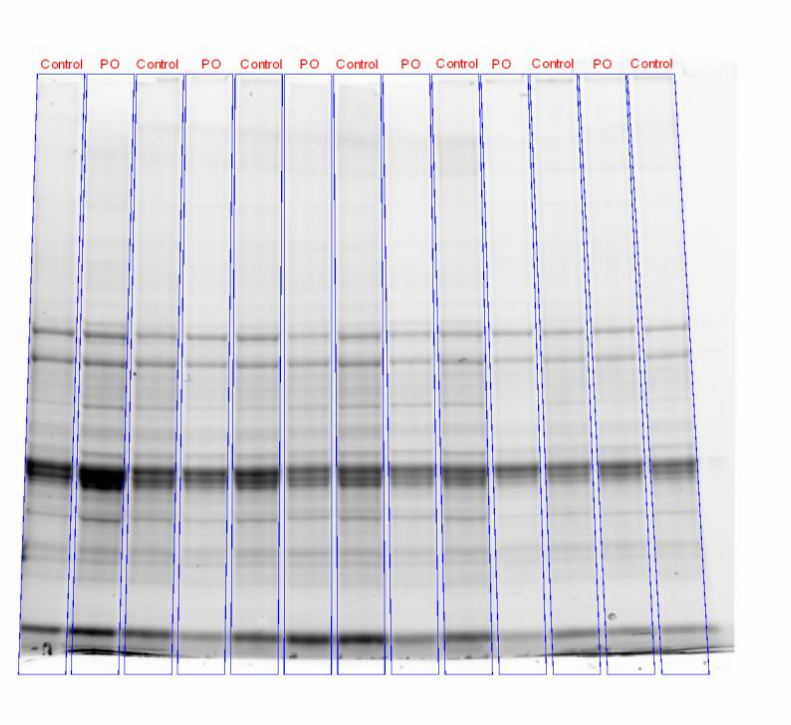


**Figure 6A** - ST-2 – 29 kDa

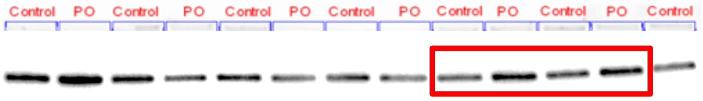


**Figure 6F** - IL-33 – 30 kDa

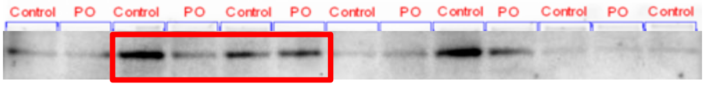


**Figure 6G** - MyD88 – 33 kDa

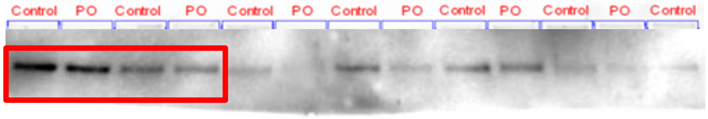


**Figure 6G** - TRAF6 – 60 kDa

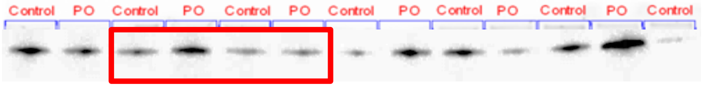


**Figure 6G** - AP-1 – 39 kDa

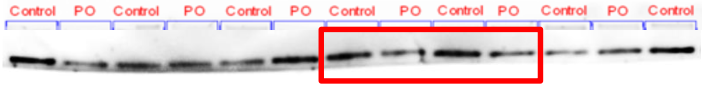


**Supplemental Figure 11**

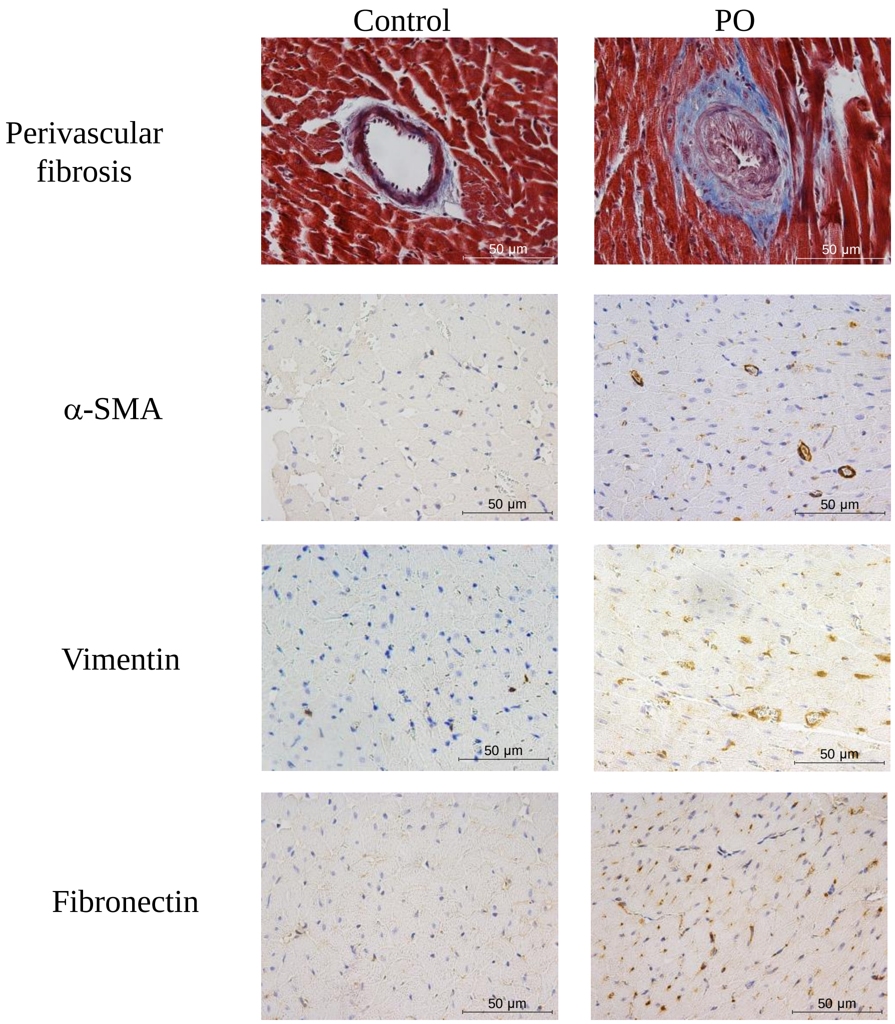


**Supplemental Figure 12**
